# Supplementary material for: Structure of the human heparan-α-glucosaminide N-acetyltransferase (HGSNAT)
Source: bioRxiv. 2024 Jun 12:2023.10.23.563672. Preprint. [Version 3] doi: 10.1101/2023.10.23.563672 (PMC10634761; doi:10.1101/2023.10.23.563672)
Supplement: 1 [file NIHPP2023.10.23.563672V3-supplement-1.pdf]

bioRxiv preprint doi: https://doi.org/10.1101/2023.10.23.563672; this version posted June 12, 2024. The copyright holder for this preprint (which was not certified by peer review) is the author/funder, who has granted bioRxiv a license to display the preprint in perpetuity. It is made available under aCC-BY 4.0 International license.

Supplementary file

Structure of the human heparan-α-glucosaminide *N*-acetyltransferase (HGSNAT)

Vikas Navratna, Arvind Kumar, Jaimin K. Rana, and Shyamal Mosalaganti

bioRxiv preprint doi: https://doi.org/10.1101/2023.10.23.563672; this version posted June 12, 2024. The copyright holder for this preprint (which was not certified by peer review) is the author/funder, who has granted bioRxiv a license to display the preprint in perpetuity. It is made available under aCC-BY 4.0 International license.

Supplementary figures

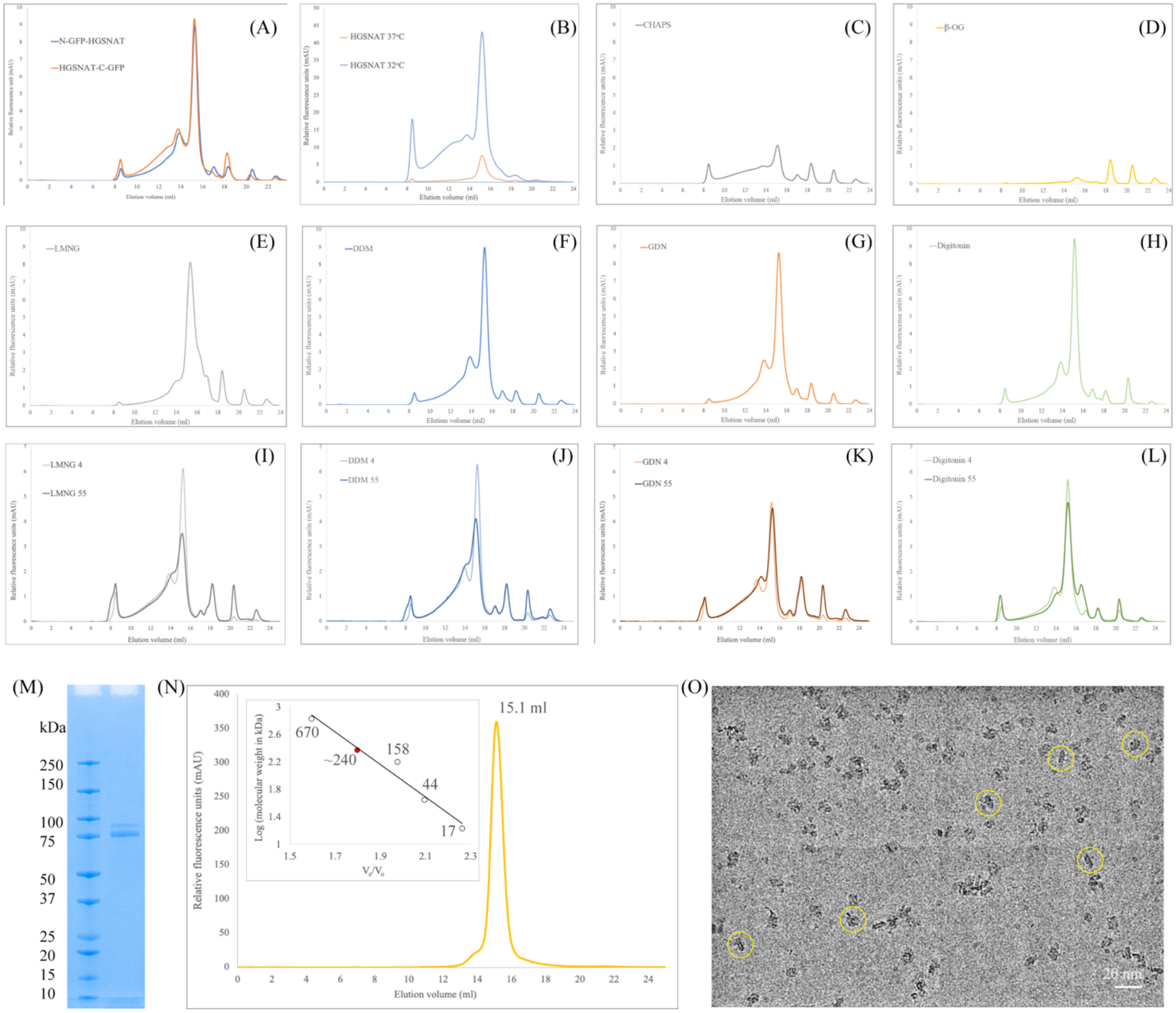

Figure S1: Purification of HGSNAT

(A) Comparison of expression of N- and C-terminal GFP fusions of HGSNAT in HEK293S GnTI$^-$ cell lysates, solubilized in 1% DDM. **(B)** Comparison of relative overexpression of N-GFP-HGSNAT in cultures grown at 37°C and 32°C, post-transduction. **(C-H)** Relative solubility and homogeneity comparison in 1% of CHAPS, β-OG, LMNG, DDM, GDN, and digitonin, respectively, prepared in 25 mM Tris-HCl, pH 7.5, 200 mM NaCl, 1 mM PMSF, 0.8 µM aprotinin, 2 µg/mL leupeptin, and 2 µM pepstatin A. **(I-L)** Comparison of relative thermal stability of

bioRxiv preprint doi: https://doi.org/10.1101/2023.10.23.563672; this version posted June 12, 2024. The copyright holder for this preprint (which was not certified by peer review) is the author/funder, who has granted bioRxiv a license to display the preprint in perpetuity. It is made available under aCC-BY 4.0 International license.

detergent solubilized HGSNAT in 1% of LMNG, DDM, GDN, and digitonin respectively. Samples analyzed after heat treatment at 55°C for 15 min have been marked with a suffix 55, and samples stored in cold room are marked with a suffix 4. **(M)** SDS-PAGE (12%) showing purity and monomeric molecular weight of HGSNAT. Although, monomeric molecular weight is ~ 100 kDa, the full-length GFP fusion of HGSNAT, like most eukaryotic membrane proteins, displays anomalous electrophoretic mobility and runs around 75 kDa. **(N)** Intrinsic tryptophan fluorescence size-exclusion chromatogram of purified HGSNAT analyzed on Superose 6 Increase 10/300 GL column at 0.5 ml/min flowrate in LMNG-based FSEC running buffer. Red dot on the standard plot (log of protein molecular weight (kDa) vs. ratio of the elution volume to the void volume (V_e/V_o) indicates that recombinant N-GFP-HGSNAT elutes at 15.1 ml corresponding to a dimer of ~ 240 kDa. **(O)** Representative micrograph imaged on Titan Krios using UltrAuFoil holey-gold 300 mesh 1.2/1.3 μm grid of vitrified N-GFP-HGSNAT at 0.9 mg/ml. The protein (yellow circles) distribution on grids, along with SDS-PAGE and size-exclusion chromatogram shows a monodisperse sample preparation.

bioRxiv preprint doi: https://doi.org/10.1101/2023.10.23.563672; this version posted June 12, 2024. The copyright holder for this preprint (which was not certified by peer review) is the author/funder, who has granted bioRxiv a license to display the preprint in perpetuity. It is made available under aCC-BY 4.0 International license.

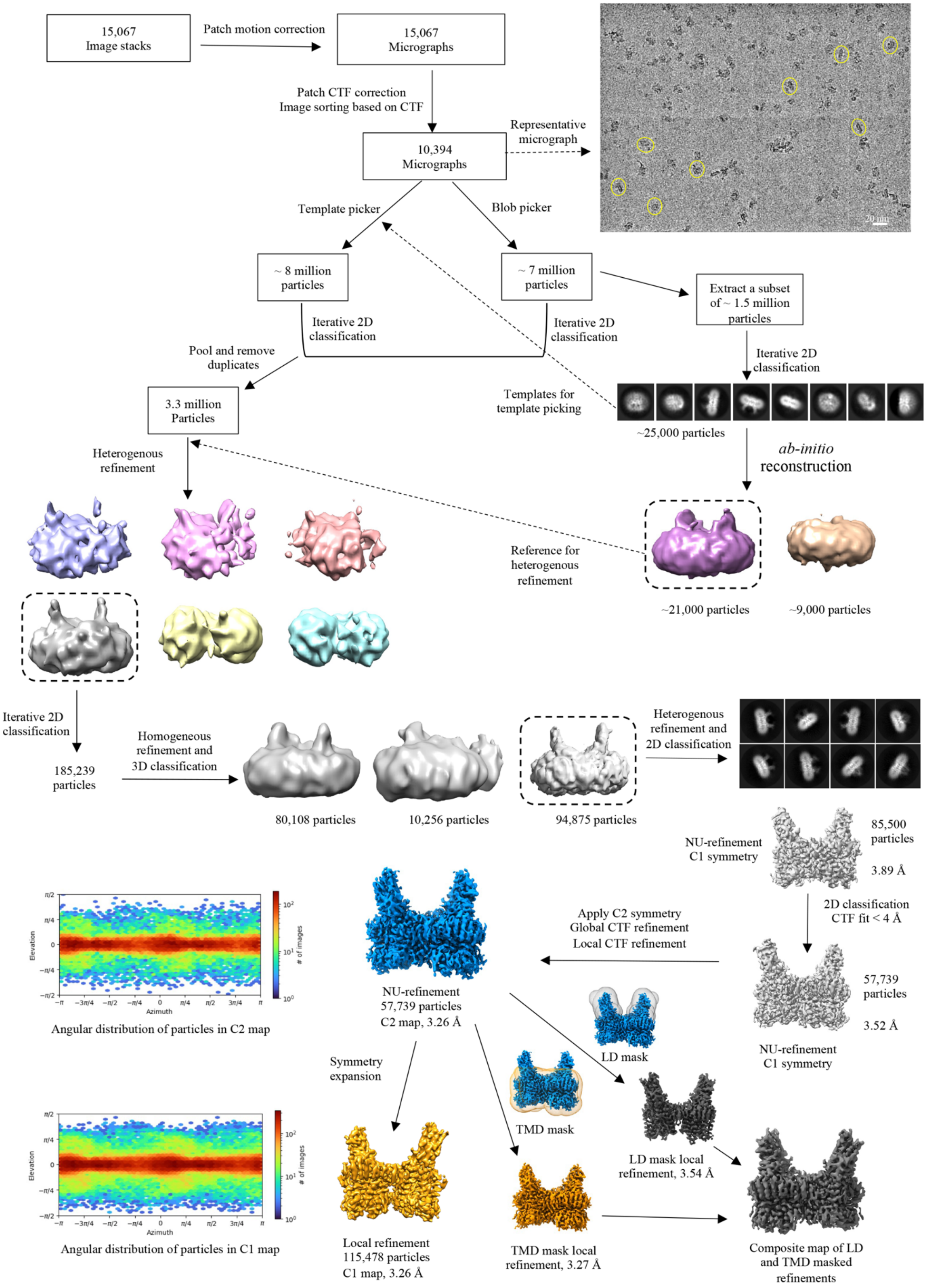

bioRxiv preprint doi: https://doi.org/10.1101/2023.10.23.563672; this version posted June 12, 2024. The copyright holder for this preprint (which was not certified by peer review) is the author/funder, who has granted bioRxiv a license to display the preprint in perpetuity. It is made available under aCC-BY 4.0 International license.

Figure S2: Cryo-EM data processing workflow

The data was entirely processed in cryoSPARC. A representative motion-corrected micrograph with single HGSNAT particles (yellow circles) is highlighted. A subset of (1.5 million) particles picked by blob picker were extracted and cleaned by 2D classification to generate 2D templates for template-based picking and an ab-initio volume to be used as reference input for subsequent heterogenous refinement jobs (dashed arrows). Particles picked using template picker and blob picker were individually cleaned by 2D classification to remove obvious junk particles and then were pooled and duplicates were removed for sorting by heterogenous refinement, and iterative 2D and 3D classification. Throughout the processing workflow classes with most well-resolved luminal domain was used as input references for the subsequent steps of processing (highlighted by a dashed boxes). A resultant stack of 85500 particles (representative 2D classes highlighted) was further cleaned up based on CTF fit (<4 Å) to end up with a final particle stack of about 57000 particles. C2 symmetry was applied at this stage and non-uniform and CTF refinements were performed to yield a C2 map at 3.26 Å. This map was used for model building and analyzing the structure of HGSNAT. A C1 map was generated by symmetry expansion of the final particle stack followed by local refinement to compare the quality of data with and without C2 symmetry application. Local refinements with masks focused on LD and TMD domain were performed separately, and a composite map was generated by combining these local refined maps to improve the density in these regions. The composite map was only used to finalize the fit of LBD side chains in the final model.

bioRxiv preprint doi: https://doi.org/10.1101/2023.10.23.563672; this version posted June 12, 2024. The copyright holder for this preprint (which was not certified by peer review) is the author/funder, who has granted bioRxiv a license to display the preprint in perpetuity. It is made available under aCC-BY 4.0 International license.

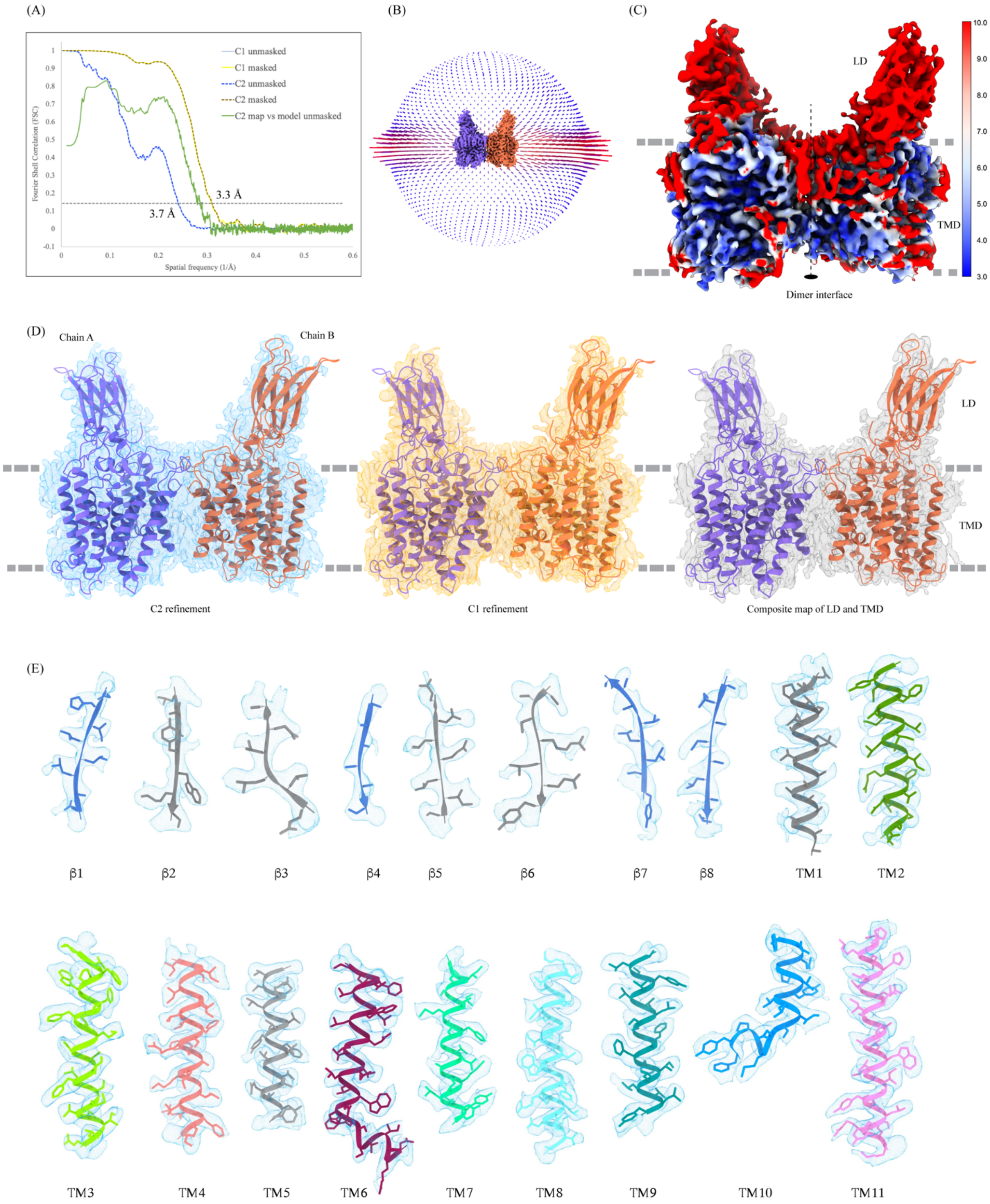

Figure S3: Cryo-EM data quality, reconstruction, and model building

bioRxiv preprint doi: https://doi.org/10.1101/2023.10.23.563672; this version posted June 12, 2024. The copyright holder for this preprint (which was not certified by peer review) is the author/funder, who has granted bioRxiv a license to display the preprint in perpetuity. It is made available under aCC-BY 4.0 International license.

(A) FSC curves for cross-validation. The final masked HSGNAT (C1: light yellow; C2: dark yellow dashed) and unmasked (C1: light blue; C2: dark blue dashed) refinement maps. Model vs. final C2 map unmasked (green). Gray and black dashed lines indicate FSC=0.143 and FSC=0.5 thresholds respectively. FSC curves were calculated using Mtriage in Phenix. **(B)** Angular distribution of particles used in the final reconstruction. **(C)** C2 map colored by estimated local resolution. **(D)** HGSNAT modelled by ModelAngelo into the C2 map (blue). The fit of the same model in C1 (orange) and composite map (gray) of LD and TMD created in ChimeraX. All maps are displayed at level 0.21 in ChimeraX. **(E)** Cryo-EM density of all the secondary structure elements, β1-β8 and TMs 1-11, shown in light blue (display level between 0.18-0.25 in ChimeraX). Side chains for almost all the elements could be modeled unambiguously into the density. At places with missing density, the side chains were trimmed to Cβ.

bioRxiv preprint doi: https://doi.org/10.1101/2023.10.23.563672; this version posted June 12, 2024. The copyright holder for this preprint (which was not certified by peer review) is the author/funder, who has granted bioRxiv a license to display the preprint in perpetuity. It is made available under aCC-BY 4.0 International license.

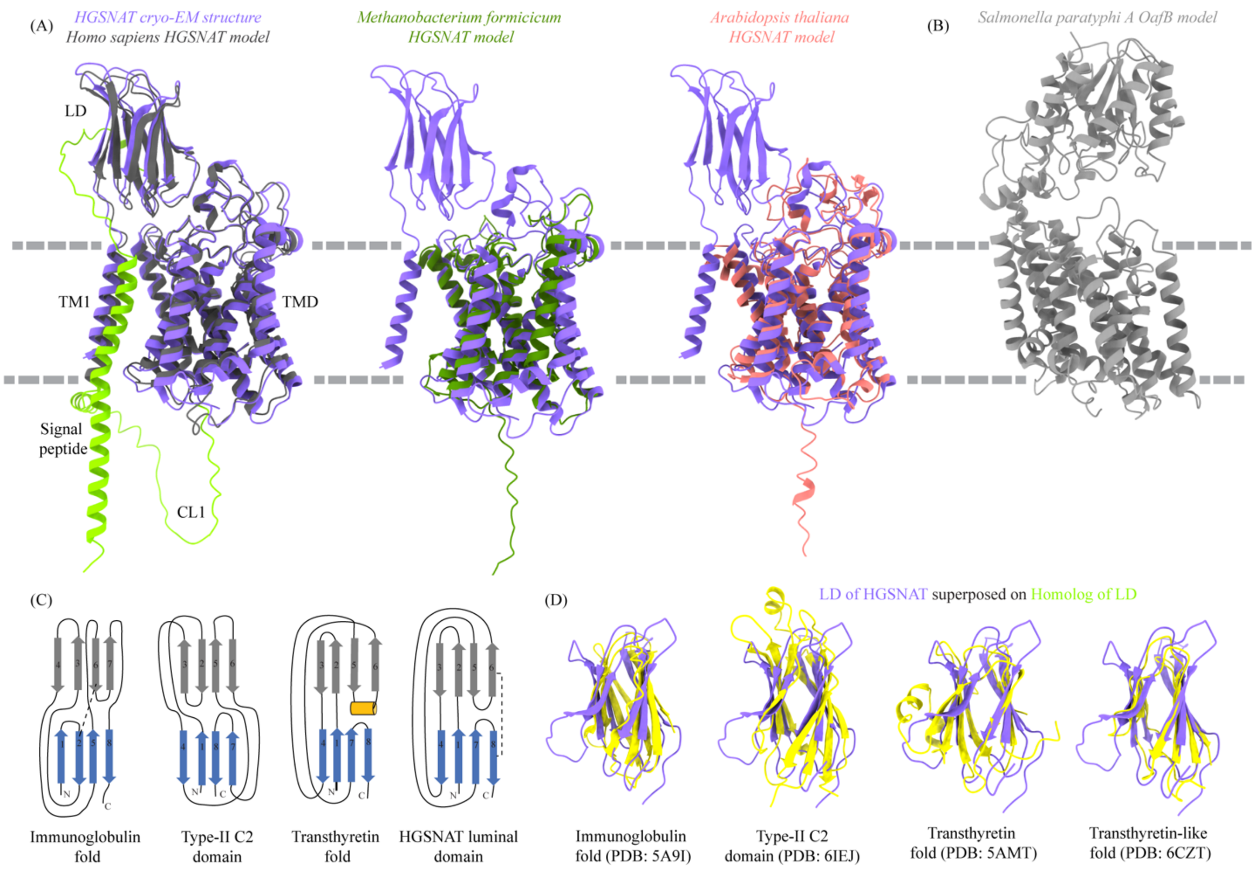

Figure S4: Homologs of HGSNAT

(A) Superposition of HGSNAT cryo-EM structure (purple) with the AlphaFold models of human (Uniprot: Q68CP4, dark gray), *Methanobacterium formicicum* (Uniprot: K2QAW2, green), and *Arabidopsis thaliana* (Uniprot: A0A5S9Y8V3, pink) HGSNATs. Cα RMSDs of the superpositions are 1.34 Å, 1.17 Å, and 1.13 Å respectively, suggesting a conserved HGSNAT fold across different kingdoms. AlphaFold model of HGSNAT shown here is of isoform 1, that has extra 28 residues on the N-term as compared to isoform 2. The structure is of isoform 2. The cryo-EM density did not allow modeling of residues upstream of β1 on the N-terminus and CL1, which have been highlighted yellow in the AlphaFold model. **(B)** AlphaFold model of acetyltransferase model of *Salmonella paratyphi* A OafB, an O-antigen modifying transmembrane acetyltransferase

bioRxiv preprint doi: https://doi.org/10.1101/2023.10.23.563672; this version posted June 12, 2024. The copyright holder for this preprint (which was not certified by peer review) is the author/funder, who has granted bioRxiv a license to display the preprint in perpetuity. It is made available under aCC-BY 4.0 International license.

of the ATAT family within the TmAT superfamily (Uniprot: A0A0H2WM30). Despite predicted to be in the same superfamily as HGSNAT, a meaningful alignment and similarity to HGSNAT was not observed, highlighting the diversity of membrane bound acetyltransferases. **(C)** Comparison of topologies of immunoglobulin (Ig) fold, type-II C2 domain, and transthyretin fold with LD of HGSNAT. Strands in two sheets are colored blue and gray and conserved helical turn in transthyretin fold is shown in orange. Conserved disulfides are shown as dashed lines. **(D)** Superposition of structures of Ig fold (PDB: 5A9I), type-II C2 domain (PDB: 6IEJ), transthyretin fold (PDB: 5AMT), and transthyretin-like domain (PDB: 6CZT) onto LD of HGSNAT. Based on the topology and structure superposition, it appears that LD is transthyretin-like domain.

bioRxiv preprint doi: https://doi.org/10.1101/2023.10.23.563672; this version posted June 12, 2024. The copyright holder for this preprint (which was not certified by peer review) is the author/funder, who has granted bioRxiv a license to display the preprint in perpetuity. It is made available under aCC-BY 4.0 International license.

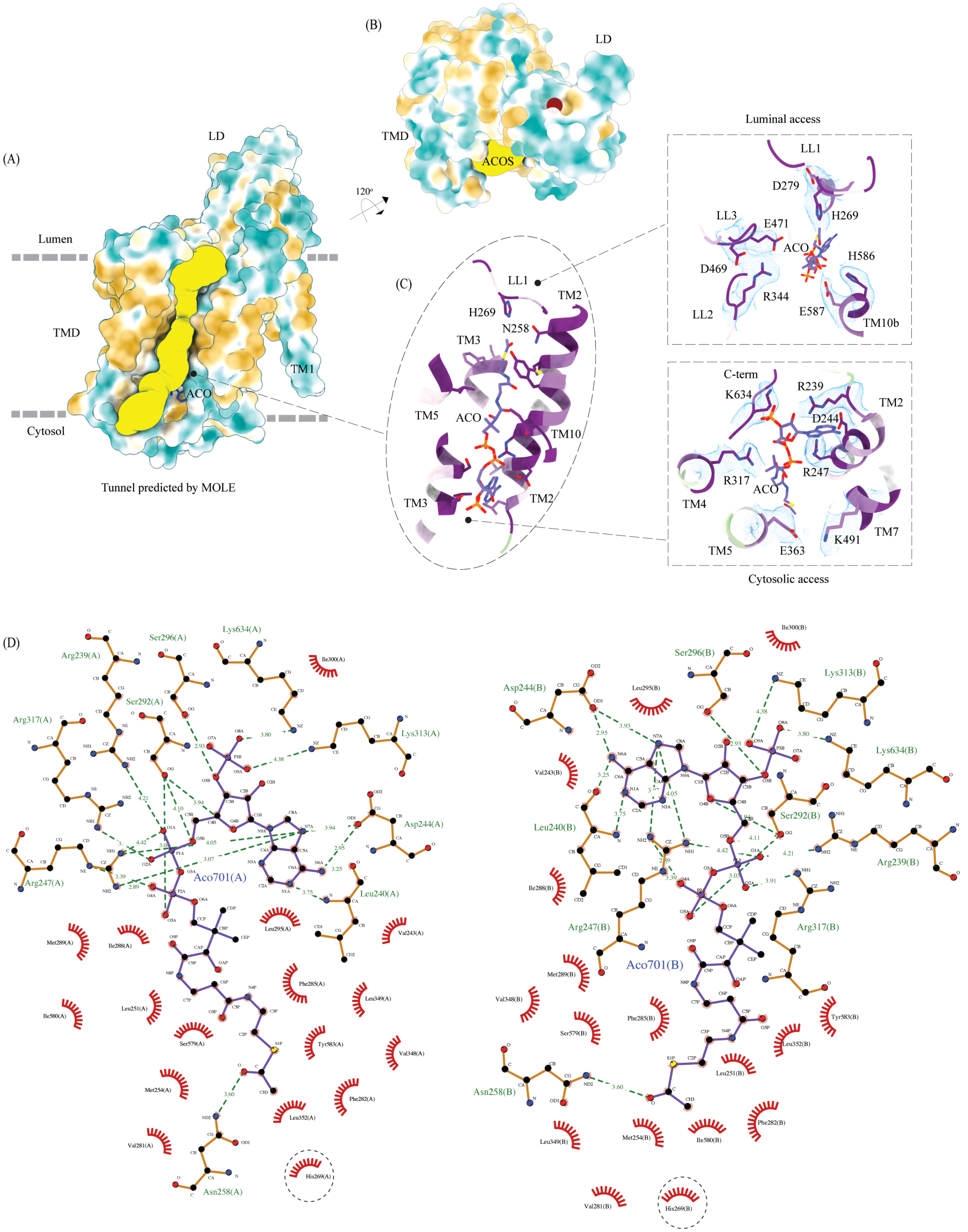

Figure S5: Ligand binding sites of HGSNAT

bioRxiv preprint doi: https://doi.org/10.1101/2023.10.23.563672; this version posted June 12, 2024. The copyright holder for this preprint (which was not certified by peer review) is the author/funder, who has granted bioRxiv a license to display the preprint in perpetuity. It is made available under aCC-BY 4.0 International license.

(A) Surface representation of HGSNAT (chain A), with hydrophobic and hydrophilic amino acids colored in orange and cyan respectively. Predicted acetyl-CoA access tunnel (yellow) by MOLEonline with a probe radius of ~ 1.5 Å (Pravda *et al,* 2018). ACO bound at HGSNAT is shown in blue. It is apparent that the nucleoside head group and the acetyl group interact with hydrophilic residues and the pantothenate group is supported by hydrophobic residues. **(B)** Ligand binding site on LD (maroon sphere) predicted by DeepSite (Jimenez *et al,* 2017) **(C)** ACOS color coded based on the evolutionary sequence conservation scores obtained from ConSurf server. In the insets are the integral salt-bridges of the luminal (top) and cytosolic (bottom) access of ACOS. The cryo-EM density for the salt-bridges is shown in blue (display level 0.22 of the C2 refine map in ChimeraX). **(D)** 2D depiction of the network of interactions of ACO modeled at chain A (left) and chain B (right) with HGSNAT residues that lie <4.5 Å away from ACO, generated in LigPlot+. Hydrogen bonds are depicted by dashed lines, and residues that are involved in hydrogen bonds with ACO are shown as ball & stick models. Non-bonded contacts are indicated as eye lashes. The predicted active site H269 is highlighted by dashed circle. In our structure N258 forms weak hydrogen bonds with the acetyl group of ACO. We believe that N258 holds onto ACO until H269 is protonated and ready for catalysis.

bioRxiv preprint doi: https://doi.org/10.1101/2023.10.23.563672; this version posted June 12, 2024. The copyright holder for this preprint (which was not certified by peer review) is the author/funder, who has granted bioRxiv a license to display the preprint in perpetuity. It is made available under aCC-BY 4.0 International license.

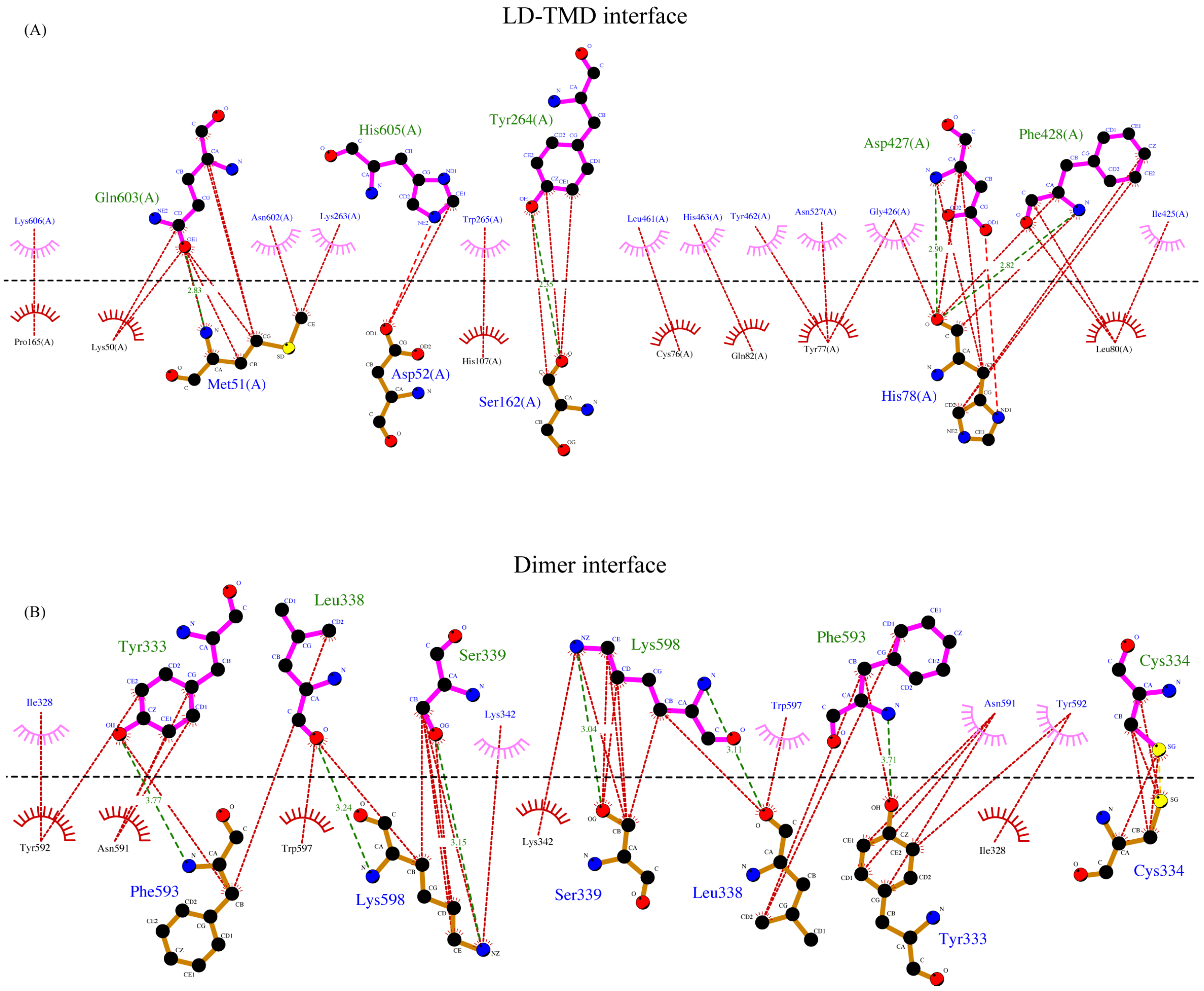

Figure S6: Interactions at LD-TMD and dimer interface

A 2D depiction of network of interactions (<4.5 Å) between residues at the LD-TMD interface (top, **A**) and at the dimer interface (bottom, **B**) generated in LigPlot+. Hydrogen bonds are shown as dashed lines with bond distance. Nonbonded and hydrophobic interactions are shown as dotted lines. Residues involved in nonbonded interactions are displayed as eye lashes. Chain A and chain B residues are shown in blue and orange. Dashed black line indicates the interface. The sulfurs of involved in disulfide bond at the dimer interface are highlighted in yellow.

bioRxiv preprint doi: https://doi.org/10.1101/2023.10.23.563672; this version posted June 12, 2024. The copyright holder for this preprint (which was not certified by peer review) is the author/funder, who has granted bioRxiv a license to display the preprint in perpetuity. It is made available under aCC-BY 4.0 International license.

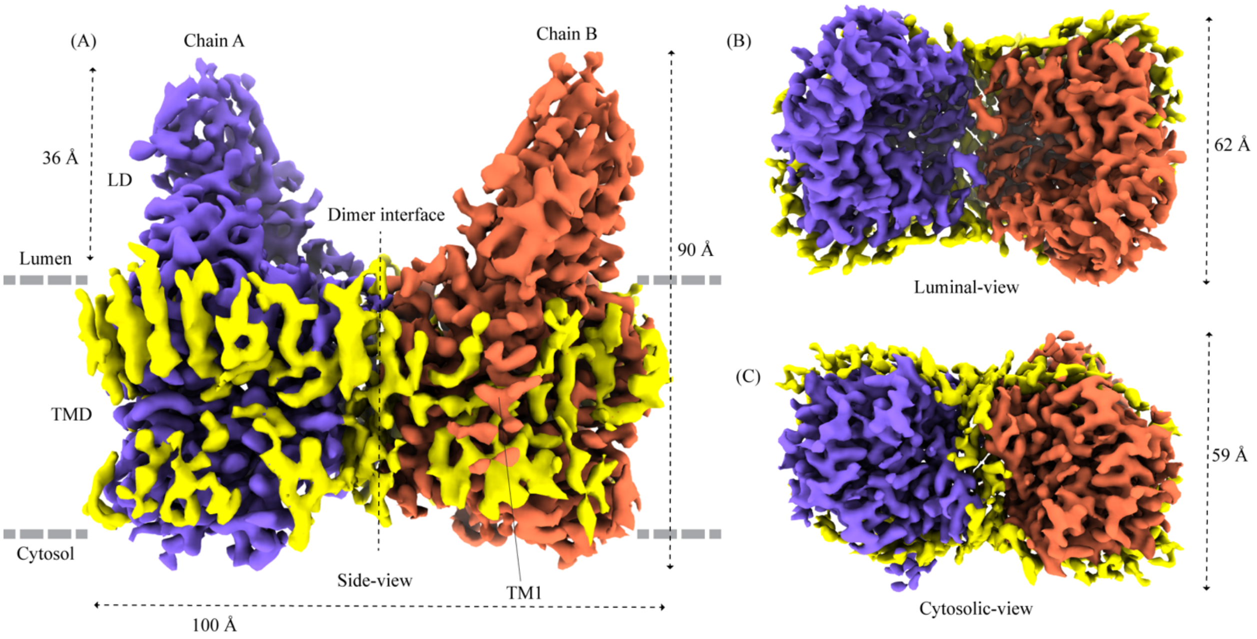

Figure S7: Lipids and detergent in the structure

Ordered density observed in our final cryo-EM map that did not account for protein and ligand has been displayed as yellow density (display level 0.22 of the C2 refine map in ChimeraX) in side-view **(A)**, luminal-view **(B)**, and cytosolic-view **(C)**. We believe these are ordered lipids and detergent molecules that interact with hydrophobic patches of the protein. Towards the cytosolic side (C) we find lipid/detergent density between the two protomers, forming a partition between two ACOSs. Chain A and chain B are shown in purple and orange. Dashed line indicates dimer interface.

bioRxiv preprint doi: https://doi.org/10.1101/2023.10.23.563672; this version posted June 12, 2024. The copyright holder for this preprint (which was not certified by peer review) is the author/funder, who has granted bioRxiv a license to display the preprint in perpetuity. It is made available under aCC-BY 4.0 International license.

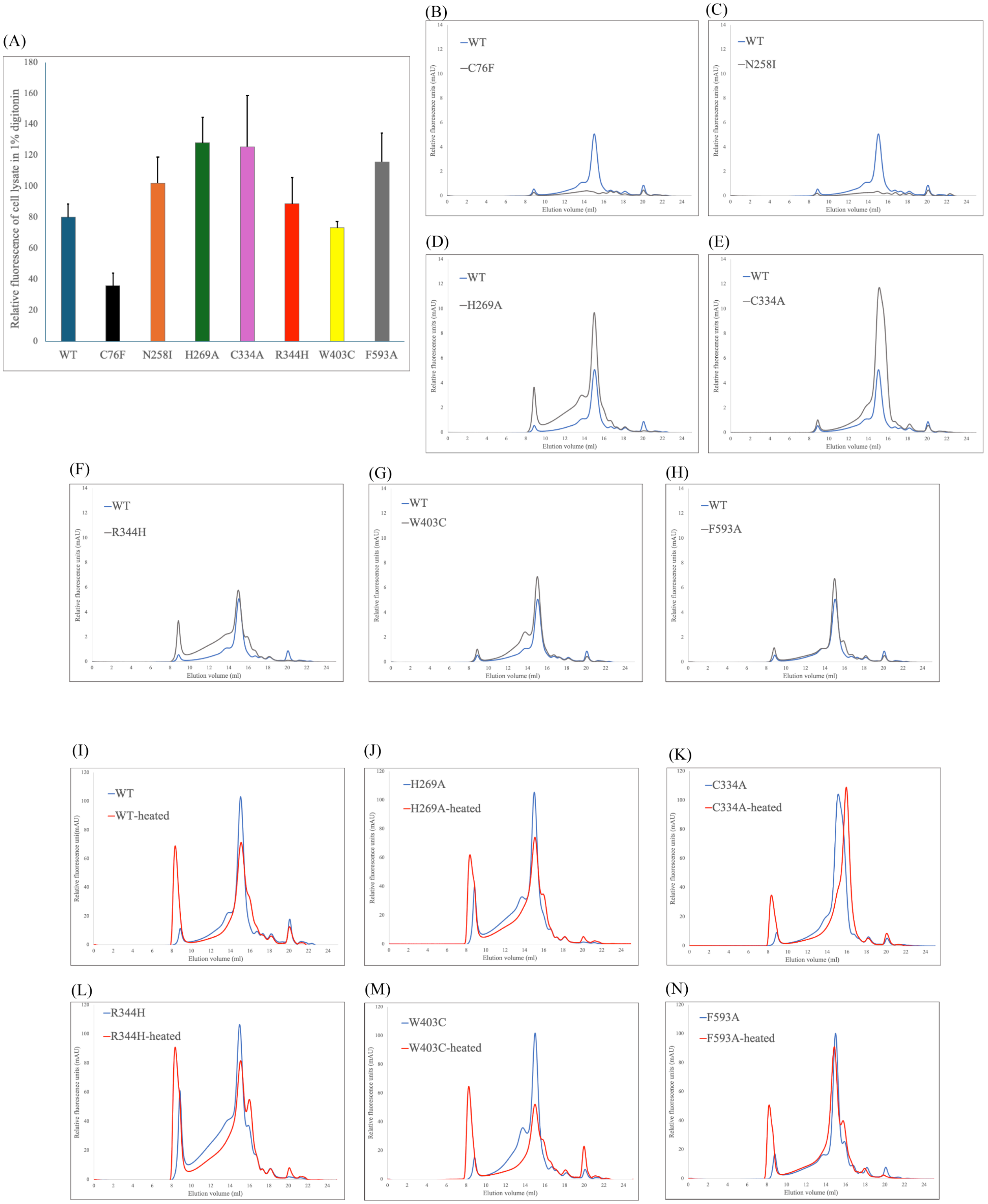

Figure S8: Expression and stability of HGSNAT mutants

bioRxiv preprint doi: https://doi.org/10.1101/2023.10.23.563672; this version posted June 12, 2024. The copyright holder for this preprint (which was not certified by peer review) is the author/funder, who has granted bioRxiv a license to display the preprint in perpetuity. It is made available under aCC-BY 4.0 International license.

(A) A comparison of relative protein expression indicated by total GFP fluorescence in 100,000 HEK293S GnTI- cells expressing HGSNAT and its mutants. **(B-H)** A comparison of FSEC chromatograms of the HGSNAT mutants (gray chromatograms) with WT HGSNAT (blue chromatogram). C76F and N258I mutants show no peak at HGSNAT dimer position, and the remaining mutants' peak position is same as dimeric WT HGSNAT. **(I-N)** Relative stability of HGSNAT mutants analyzed by FSEC. To estimate relative stability of mutants, the solubilized mutant cell lysates were heated at 65°C for 15 min (red chromatograms) and the loss of HGSNAT peak in the resultant chromatograms were compared with non-heated samples (blue chromatograms). C334A, the mutant which breaks the disulfide at the dimer interface, results in a monomeric HGSNAT peak upon heating, while all other mutants retain their dimeric status.

bioRxiv preprint doi: https://doi.org/10.1101/2023.10.23.563672; this version posted June 12, 2024. The copyright holder for this preprint (which was not certified by peer review) is the author/funder, who has granted bioRxiv a license to display the preprint in perpetuity. It is made available under aCC-BY 4.0 International license.

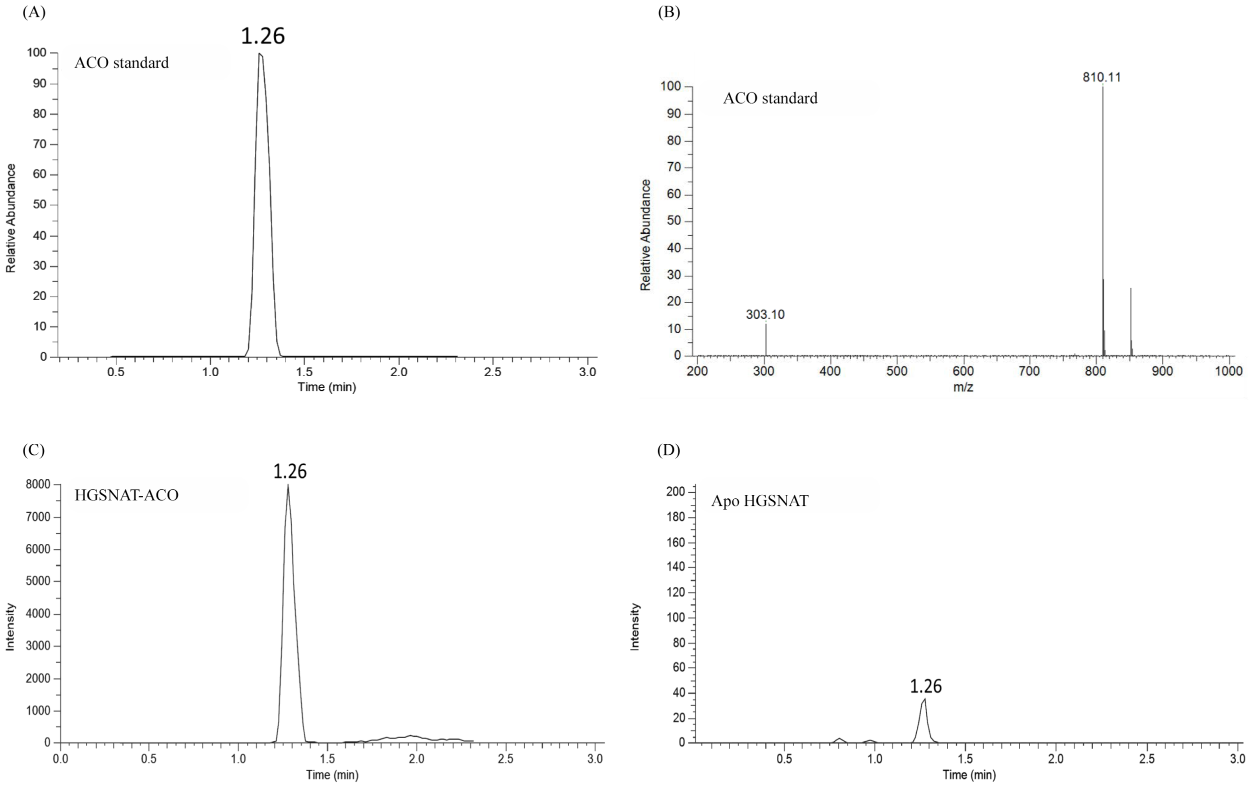

Figure S9: LC-MS analysis of purified HGSNAT

(A) LC profile and **(B)** MS/MS spectrum of acetyl-CoA (ACO) standard, showing the retention time (1.26 min), and precursor (810.1 m/z) and product (303.1 m/z) peaks in single reaction monitoring mode, respectively. **(C)** and **(D)** show relative LC peak intensities of endogenously bound ACO identified in purified HGSNAT before and after dialysis of the membranes respectively.

bioRxiv preprint doi: https://doi.org/10.1101/2023.10.23.563672; this version posted June 12, 2024. The copyright holder for this preprint (which was not certified by peer review) is the author/funder, who has granted bioRxiv a license to display the preprint in perpetuity. It is made available under aCC-BY 4.0 International license.

Supplementary tables and legends

Table S1: Homologs of TMD and LD of HGSNAT found in a Dali search

PDB ID	RMSD (Å)	Number of equivalent residues	Total number of residues	% Identity	Protein
Homologs of TMD					
5LNK	5.8	84	175	17	Mitochondrial complex 1, 51 kDa subunit
5CE3	4.3	93	598	16	Actin
6B2Z	9.1	87	249	15	Mitochondrial ATP synthase subunit C
6HV6	4	66	293	15	PatoxP toxin
6KKK	5.9	133	380	14	Sugar efflux transporter SotB
Homologs of LD					
6CZT	2	81	82	15	AlgF, alginate biosynthesis protein
6IXH	3.1	87	123	14	Type VI secretion system core complex TSSJ
6G7G	3.1	88	115	13	S-protein homolog (SPH) 15
5A9I	2.3	95	194	12	ECD of PepT2
5AMT	3.7	84	105	12	Intracellular growth locus E (IglE)

Dali (**D**istance **Ma**trix **Ali**gnment) web server was used to search the existing database of known structures to find homologs of HGSNAT (Holm *et al,* 2023). The poor % identity (<20%) and low % sequence alignment suggests that there are no available structures of homologs of HGSNAT. Low mean RMSD of hits obtained using LD of HGSNAT as input suggests that LD is like some of the existing β-sandwiches, but TMD of HGSNAT is a novel fold.

bioRxiv preprint doi: https://doi.org/10.1101/2023.10.23.563672; this version posted June 12, 2024. The copyright holder for this preprint (which was not certified by peer review) is the author/funder, who has granted bioRxiv a license to display the preprint in perpetuity. It is made available under aCC-BY 4.0 International license.

Table S2: List of HGSNAT mutations implicated in MPS IIIC

Mutation	Total energy (kcal/mol)	Region of the protein
G423W	41.0	LD-TMD interface
G424V	18.8	
G424S	11.8	
G262R	7.5	
C76F	5.0	
N273K	-0.6	
G133A	-1.4	
P283L	3.0	Catalytic core
R344H	2.0	
R344C	2.0	
E471K	1.4	
N258I	-0.1	
G486E	17.9	Scaffold domain
M482K	3.2	
S518F	2.5	
W403C	2.2	
A489E	1.1	
K523Q	0.9	
S539C	0.7	
S541L	-0.2	
V481L	-0.3	
L445P	5.9	Other regions
L113P	4.9	
L137P	3.4	
A54V	2.9	
A615T	2.3	
Y627C	1.4	
D562V	1.2	
P237Q	0.1	
P571L	-0.2	
G173D	-0.4	
G173E	-0.9	

FoldX web server was used to predict relative mutant stability (Schymkowitz *et al,* 2005). Positive total energy value indicates destabilization, with greater values meaning lower stability. Nonsense mutations indicated in the figure 4 as black were not included in FoldX calculations. Polymorphisms are italicized. All other mutants listed are missense mutations (Canals *et al,* 2011;

bioRxiv preprint doi: https://doi.org/10.1101/2023.10.23.563672; this version posted June 12, 2024. The copyright holder for this preprint (which was not certified by peer review) is the author/funder, who has granted bioRxiv a license to display the preprint in perpetuity. It is made available under aCC-BY 4.0 International license.

Fan *et al,* 2006; Fedele & Hopwood, 2010; Feldhammer *et al,* 2009a; Feldhammer *et al,* 2009b; Hrebicek *et al,* 2006; Huizing & Gahl, 2020).
